# Real‐world Israeli single institution experience with PET‐PSMA for staging of patients with clinically staged localized prostate carcinoma

**DOI:** 10.1002/cnr2.1386

**Published:** 2021-05-02

**Authors:** Yonaton Zarbiv, Yehudit Peerless, Marc Wygoda, Marina Orevi, Karen Meir, Ofer N. Gofrit, Vladimir Yutkin, Stephen Frank

**Affiliations:** ^1^ Sharett Institute of Oncology Hadassah‐Hebrew University Medical Center Jerusalem Israel; ^2^ Department of Nuclear Medicine Hadassah‐Hebrew University Medical Center Jerusalem Israel; ^3^ Department of Pathology Hadassah‐Hebrew University Medical Center Jerusalem Israel; ^4^ Department of Urology Hadassah‐Hebrew University Medical Center Jerusalem Israel

**Keywords:** clinical observations, PET PSMA, prostate cancer

## Abstract

**Background:**

A recent prospective trial, the proPSMA study, showed superior specificity and sensitivity of Positron emission tomography (PET) ‐ Prostate‐specific membrane antigen (PSMA) imaging compared standard Computerized tomography (CT) and bone scan for staging of recently diagnosed high‐risk local prostate carcinoma for curative intent treatment.

**Aim:**

To share our experience with false‐positive PET PSMA scans in newly diagnosed intermediate‐risk prostate cancer.

**Methods and results:**

Here, we report a series of eight patients who underwent systemic staging using PET‐PSMA with false‐positive results who were ultimately treated with definitive radiation or surgery. Of the eight patients, two patients were diagnosed with favorable intermediate disease, four with unfavorable intermediate risk, and two with high‐risk disease. Seven of eight were shown to have false‐positive bone uptake, one patient had uptake in lung nodules. Three patients underwent bone biopsy and proven benign. The rest of the patients were proven as non‐metastatic radiologically by repeat PSMA, CT, or Magnetic resonance imaging (MRI). All subsequently preceded to definitive localized treatment and remain disease free as of this study.

**Conclusion:**

This study emphasizes the importance of prudent clinical judgment when utilizing this highly sensitive imaging technique.

## 1 INTRODUCTION

Prostate cancer is the most common cancer in men worldwide and is second only to lung cancer in cancer mortality among men.^1^ According to the Surveillance, Epidemiology and End Results (SEER) database, 77% present with localized disease, 13% with regional lymph node involvement, and 6% with metastatic disease.^2^ The current NCCN guidelines recommend bone scan as initial staging in all patients with high‐risk disease defined as T3/T4 or PSA >20 or Gleason 8‐10 and intermediate risk defined as T2b and T2c or Gleason 4 + 3 or PSA >10.^3^ Similarly, American Society of Clinical Oncology (ASCO) guidelines recommend bone imaging for all high risk and unfavorable intermediate risk patients as previously defined.^4^ PET PSMA has been shown to have higher sensitivity for detection of bone metastases as compared to bone scan.^5^ PET PSMA has also been shown to have high specificity for lymph node involvement although only moderate sensitivity.^6^ A recent prospective randomized trial compared conventional imaging with CT and bone scan to imaging with PET PSMA in newly diagnosed high‐risk prostate cancer. Patients underwent crossover after initial imaging and results were analyzed to show accuracy of first‐line imaging in diagnosis of lymph node involvement and distant metastases. PET PSMA was superior to conventional imaging with respect to specificity and sensitivity and resulted in more frequent changes in treatment plan.^7^ Despite these data, it remains unclear whether low and intermediate risk patients warrant PET PSMA for staging. The benefit of improved sensitivity of PET PSMA inevitably results in false positivity,^8^ causing unnecessary investigations with potential of false upstaging and increased financial costs. In Israel, PET PSMA is in the national health care basket for newly diagnosed prostate cancer and has become the standard of care, allowing us to gain much needed real world experience. Since 2016, the Israeli health basket has included PET PSMA for initial staging of localized prostate adenocarcinoma with Gleason 7‐10 and/or PSA > 20, and or T3‐4. The inclusion of Gleason 7 in the health ministry's guidelines, (grade group 2 or 3) with no other high‐risk features allows for the systemic screening of intermediate group patients. Furthermore, patients are regularly referred to and reimbursed for initial PET PSMA scanning with only intermediate risk features often before initial evaluation by an oncologist.

We, therefore, currently scan all newly diagnosed with the above‐mentioned risk factors, and many with low‐intermediate risk features. We, therefore, have a wealth of real‐world experience of PSMA imaging in a population that does not only include high‐risk patients. From October 2019 to April 2020, we collected cases of newly diagnosed prostate cancer with positive PSMA scans in patients who clinically were suspected to have local disease only. We report here a series of patients at our institution that underwent imaging with PET PSMA, with positive findings that resulted in additional imaging studies or biopsies before reverting to the original treatment plan when the PET PSMA was deemed as having false‐positive results.

Our study was approved by local IRB.

## 2 CASE 1

**FIGURE 1:**
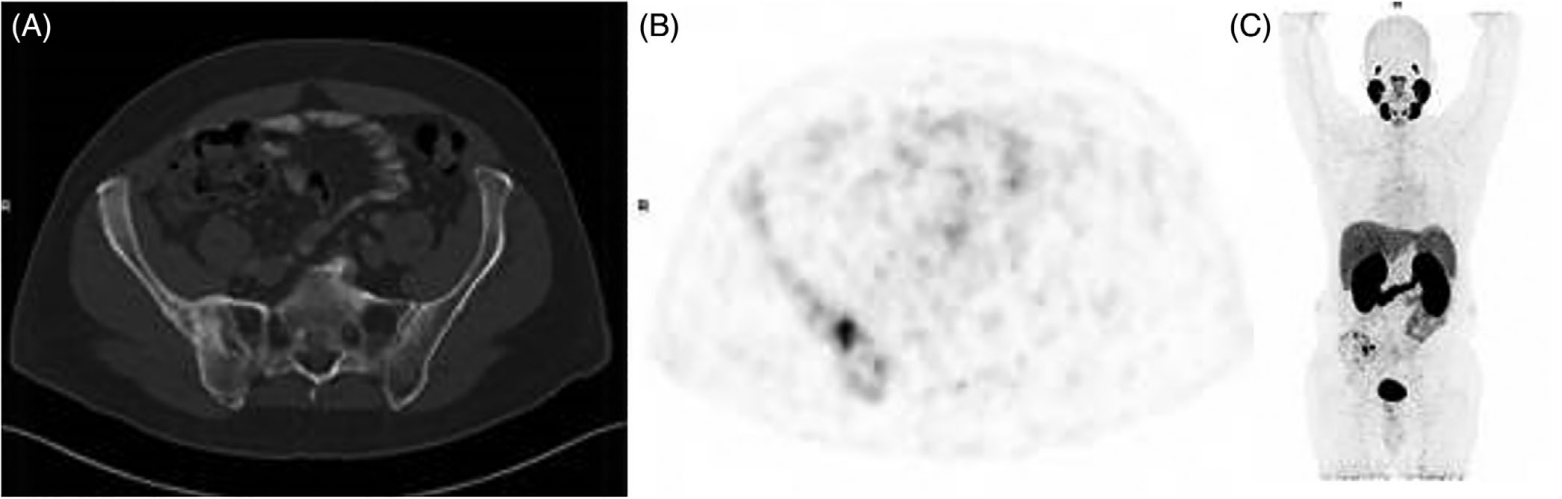
Ga^68^PSMA PET/CT of 65‐year‐old patient with known prostate cancer (Gleason 3 + 4, PSA 4.7) shows sclerotic changes in the right iliac bone A, with highly increased (SUV max 14.6) PSMA accumulation, B and C, MIP maximum intensity projection). Paget disease diagnosed on later performed biopsy

**FIGURE 2:**
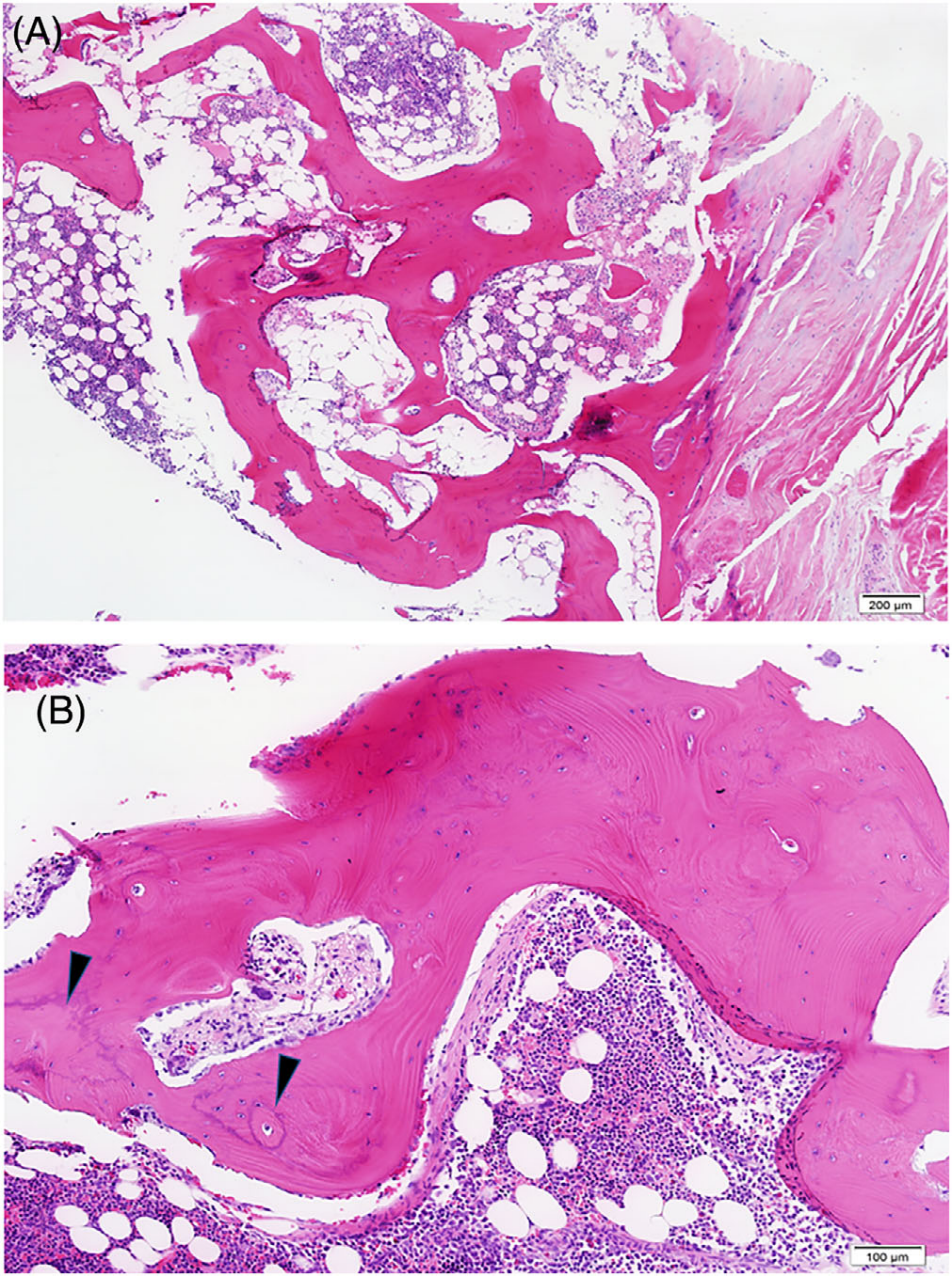
A and B, Patient 3, trephine biopsy. Note thickened irregular bony trabecula with focally prominent cement lines (darts), suspicious for Paget disease of bone. Metastatic carcinoma was not observed. NKX3.1 immunostain was negative (not shown). [hematoxylin and eosin]

A 65‐year‐old man with a personal history of diabetes and hypertension and a family history of prostate cancer in father and brother presented with nocturia. TRUS showed enlarged prostate 46 × 51 × 26 mm^3^ and PSA of 4.67. MRI of the prostate was suggestive of malignancy with left and right hypo‐intense lesions on the peripheral zone PIRADS 4 abutting the capsule with no seminal vesicle involvement with a radiological cT2cN0. MRI‐fused biopsy was performed, and pathology was positive for prostatic adenocarcinoma in up to 80% of 10/16 bilateral cores, grade group 2 with Gleason 3 + 4. This patient with clinical stage unfavorable intermediate risk was sent for a PET‐PSMA, which showed sclerotic multiple lesions with strong uptake of PSMA in the right ilium (Figure 1). Subsequent biopsy of the lesions had no evidence of malignancy. Interestingly, however, the cores of bone exhibited foci of irregular, markedly thickened trabecula, suspicious for Paget's disease of bone (Figure 2). The patient went on to receive androgen deprivation therapy and definitive radiation to the prostate.

## 3 CASE 2

**FIGURE 3:**
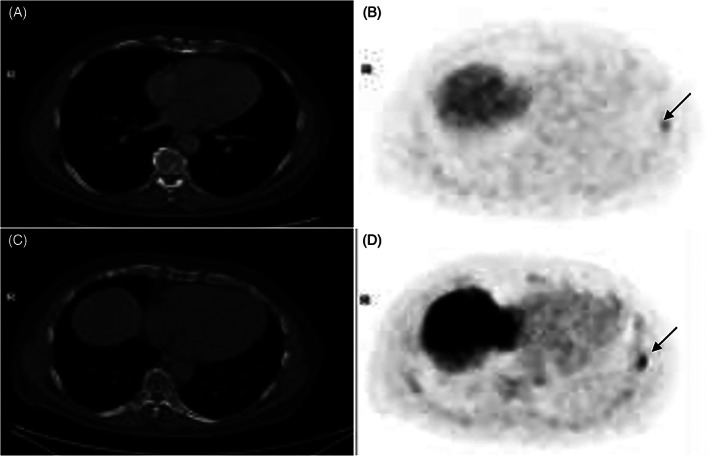
Two PET/CT scans of 72‐year‐old patient with known prostate cancer (Gleason 4 + 3, PSA 5.5) performed with F^18^PSMA (lower row) and 2 month later with Ga^68^PSMA (upper row). Focus (arrow) of increased uptake (b, d) visualized in both scans in the seventh left rib without significant interval changes (slightly less prominent intensity of uptake on the Ga^68^PSMA scan in comparison with the F^18^PSMA). Negative for prostate cancer results obtained from biopsy of this rib

A 73‐year‐old man with medical history of Wolf‐Parkinson‐White syndrome suffered from nocturia, the prostate was enlarged on digital rectal exam in the right apex with a PSA of 7. MRI of the prostate showed a focal ovular 0.4 × 0.6 CM hypo‐intense mass on T2 imaging PIRADS 4. Capsular invasion and seminal vesical involvement were not evident. Subsequent MRI fused and random biopsies showed prostatic adenocarcinoma in the right apex in 1/3 cores and the left apex in 1/3 cores, both areas grade group 3 (Gleason 4 + 3) in 5% to 10% of the cores. Clinically staged as T2c, unfavorable intermediate risk disease, a PET PSMA was interpreted as showing likely pathological uptake in the right fourth rib, left seventh rib, left humerus, and L2 vertebrae (Figure 3). Due to the low clinical suspicion of metastatic disease, we opted to repeat the scan after 2 months and delay intervention. Repeat scan showed persistent uptake in the left seventh rib. A biopsy of the site showed normal bone tissue and the patient was determined to have local disease only. He underwent radical prostatectomy 10 months prior to publication, and has no evidence of biochemical recurrence.

## 4 RESULTS

We report eight cases of suspected metastatic disease in PET‐PSMA upon initial staging which on further investigation were shown to be false positive. Two patients were initially diagnosed with favorable intermediate disease, four with unfavorable intermediate risk, and two with high‐risk disease. The most common sight of false‐positive uptake was in the bone seen in 7/8 patients, five of whom had single areas of uptake and two had multiple suspected bone metastases. One patient had high uptake of PSMA in lung nodules. Six patients were proven to be with local disease only using subsequent imaging: two had repeat PET PSMA 2 to 3 months later, two had MRI of the suspicious bone lesion, and two had CT of the bone lesion. A patient with suspicious lung nodules was shown to have stable nodules on high resolution CT compared to a CT done 1 year prior. Three patients were proven to be free of metastasis with bone biopsy including one who had both biopsy and repeat PET PSMA. All patients received definitive local therapy either with Radical Prostatectomy or ADT + definitive radiotherapy. No patients had radiological or biological evidence of recurrence as of submission of this article with an average follow up of 13 months. The results are summarized in Table 1.

**TABLE 1:** Clinical characteristics, sight of PSMA uptake and subsequent workup, treatment and follow‐up of 8 patients with PET PSMA initially suspicious of metastatic disease ultimately treated with definitive local therapy.

	Age at diagnosis	Gleason	PSA	Grade group	Clinical T stage	Risk group (NCCN)	Sight of PSMA uptake	Subsequent imaging	Biopsy	Definitive treatment	Time to follow‐up (months)
1	65	3 + 3	12.6	1	T2	Favorable intermediate	Sacrum	None	Normal bone tissue	Radiation + ADT	13
2	77	3 + 4	9.6	2	T2	Favorable intermediate	2 lesions Rt ribs	MRI of ribs‐ no lesion to biopsy	No	Radiation + ADT	9
3	65	3 + 4	4.7	2	T2	Unfavorable intermediate (80% positive cores)	Rt iliac bone	None	Paget's disease of bone	Radiation + ADT	9
4	72	4 + 3	5.5	3	T2	Unfavorable intermediate	Oligometastatic bone disease with 4 suspected metastases	Repeat PET PSMA	Normal bone tissue	Radical Prostatectomy	21
5	69	4 + 3	6.6	3	T2\T3?	Unfavorable intermediate	Rt iliac	pelvic MRI‐ no lesion to biopsy	No	Radiation + ADT	9
6	76	4 + 3	12	3	T1C	Unfavorable intermediate	Lung nodules	CT‐ stable lesions	No	Radiation + ADT	9
7	69	4 + 5	5.6	5	T2	High risk	Rt rib	Follow up PET	No	Radiation + ADT	7
8	72	4 + 4	11.7	4	T2b	High risk	Lt. rib	CT no lesion for biopsy	No	Radiation + ADT	11

Abbreviations: PSA, Prostate specific antigen; ADT, androgen deprivation therapy.

## 5 DISCUSSION

The Israeli health care system incorporating PET‐PSMA imaging as initial staging for early stage prostate cancer has allowed us to access this new modality and gain real world experience. Our center currently scans all patients with high risk and many with intermediate risk local prostate cancer, specifically Gleason 3 + 4 as well as many patients with only intermediate risk factors if the clinician requested the scan. We have performed hundreds of scans since PET‐PSMA was introduced in Israel in 2016.

PET‐PSMA has the ability to alter treatment plans for patients, allowing them to be staged upon initial diagnosis with locally advanced or metastatic disease and receive appropriate treatment. This may prevent morbidity and mortality from unnecessary or unsuitable local treatment.

However, in our experience, false‐positive scans may inaccurately upstage patients with local disease excluding them from receiving potentially lifesaving definitive local treatment. Additionally, inappropriate long‐term hormonal therapy or chemotherapy can cause potential detrimental physical and emotional side effects.

Hofman et al recently published a phase 3 randomized multicenter trial assessing the use of PET‐PSMA before curative‐intent surgery or radiotherapy (the proPSMA trial). This trial included high‐risk patients defined as having at least a PSA of 20 ng/mL, grade group 3‐5, or stage T3. The authors concluded that for this group of patients, PET‐PSMA is both more sensitive and specific than conventional imaging. This seminal study may be practice changing and result in the widespread use of PET‐PSMA in this population.^7^

In our series, five of the eight patents with false‐positive PET PSMA scans fulfilled the risk category clinical stage criteria of the ProPSMA Trial and would have been included in the study population.

However, it is important to note that a subgroup of patients in the ProPSMA trial would be considered unfavorable‐intermediate risk based on the NCCN guidelines. Therefore, one could argue that there is a benefit of staging for intermediate risk prostate cancer patients in order to find occult metastases as long as unexpected findings are confirmed with follow up. This emphasizes the need for judicious use of imaging as staging for lower risk patients, specifically patients with low PSA, T2, and grade group 2. More studies on this population are required. It is of note that even according to the liberal Israeli guidelines allowing for scanning of favorable intermediate risk with a Gleason score of 3 + 4 many lower risk patients were also scanned. It may be prudent to forgo PET‐PSMA scanning in this low‐risk population, as the risk of a false‐positive result may be unacceptably high.

This case series highlights the importance of clinical judgment, interdisciplinary decision making, appropriate allocation of costly medical resources, and occasionally aggressive procedures including biopsy to accurately stage patients. Ultimately, this distinction is critical in formulating the decision to initiate curative local treatment vs palliative systemic interventions. Importantly, the Food and Drug Administration (FDA) has recently improved PET‐PSMA for initial staging of newly diagnosed prostate cancer. Our series illustrates the importance of the proper interpretation of the results of this new modality.

## CONFLICT OF INTEREST

The authors declare no conflicts of interest.

## AUTHORS' CONTRIBUTIONS

All authors had full access to the data in the study and take responsibility for the integrity of the data and the accuracy of the data analysis. *Conceptualization*, Y.Z., Y.P., S.F.; *Methodology*, Y.Z., Y.P., S.F.; *Formal Analysis*, Y.Z., Y.P., M.O.; *Writing ‐ Original Draft*, Y.Z., Y.P.; *Writing ‐ Review & Editing*, Y.Z., Y.P.; *Visualization*, M.O., K.M.; *Data Curation*, M.W., M.O., K.M., O.G., V.Y., S.F., *Supervision*, S.F.

## ETHICAL STATEMENT

The study was conducted at the Sharret Institute of Oncology, after approval by the institutional review board (0394‐20‐HMO). Informed patient consent was waved for this study.

## Data Availability

The data that support the findings of this study are available on request from the corresponding author. The data are not publicly available due to privacy or ethical restrictions.
